# Application of Conservation Genomics to Investigate the Role of Pathogens on the Migration of Sea‐Run Brown Trout (*Salmo trutta*)

**DOI:** 10.1111/jfd.14045

**Published:** 2025-01-08

**Authors:** Robert J. Lennox, Sindre H. Eldøy, Angela D. Schulze, Kristina M. Miller, Trond Einar Isaksen, Jan G. Davidsen, Cecilie I. Nilsen, Lotte S. Dahlmo, Knut Wiik Vollset

**Affiliations:** ^1^ Laboratory for Freshwater Ecology and Inland Fisheries NORCE Norwegian Research Centre Bergen Norway; ^2^ Ocean Tracking Network, Dalhousie University Halifax Nova Scotia Canada; ^3^ Department of Natural History NTNU University Museum Trondheim Norway; ^4^ Pacific Biological Station, Fisheries and Oceans Canada Nanaimo British Columbia Canada

**Keywords:** acoustic telemetry, aquaculture, climate change, fluidigm, synergy

## Abstract

Pathogens play a key role in individual function and the dynamics of wild populations, but the link between pathogens and individual performance has rarely been investigated in the wild. Migrating salmonids offer an ideal study system to investigate how infection with pathogens affects performance given that climate change and fish farming portend increasing prevalence of pathogens in wild populations. To test for effects of pathogen burden on the performance of a migrating salmonid, we paired data from individual brown trout tagged with acoustic accelerometer transmitters and gill biopsies to investigate how pathogen infection affected whole animal activity during the spawning migration. Generalised additive models fitted to the acceleration data revealed individual and temporal variation in acceleration as expected, but also provided a significant effect of relative infection burden on acceleration. However, when linking this pathogen‐specific effect to a relevant bioenergetic change, it was evident that the effect had little impact on the exercise‐related oxygen consumption at the individual level, especially in cases where fish were not exerting high exercise activity. The results are a powerful example of how pairing non‐lethal biopsies with individual tracking technologies can be used to assess how pathogens impact fish in situ.

## 1 Introduction

Energy is a critical currency for migrating animals (Blem 1980; Klaassen 1996). Because migration entails the cessation of feeding and relatively intense activity of individuals to move towards spawning or feeding grounds, individuals must follow a strict budget to allocate energy to transport, maintenance, maturation and reproduction (Lennox et al. 2016). Performance of individuals engaging in migration will ultimately determine their fitness; the ability to have stored enough energy and to reserve enough to reach the destination is crucial. Given that energy acquisition is quite limited during migration, judicious expenditure of energy along the migration is paramount. The energy expenditure of ectothermic animals, however, depends on the external environment (Fry 1947). Biological factors that affect energy demands can have a significant negative impact on migratory species by disrupting the energy budget leading to negative fitness outcomes for fish or even premature mortality.

Infection with pathogens can reduce energy availability for animal migration (Palstra et al. 2007; Wagner et al. 2005). Infectious pathogens can cause disease, which occurs when the body's normal function is impaired; many diseases are associated with infectious agents such as viruses or bacteria. Individuals can co‐exist with their pathobiome without incurring disease, but certain conditions may favour the rapid replication of a pathogen that leads to disease and a mounting burden upon the animal leading to a state of physiological impairment. In such cases, the individual's innate and adaptive immune responses are important to fighting the further development of disease (Mokhtar et al. 2023). Mounting an immune response when challenged by a disease‐causing agent benefits the animal but also carries costs, affecting the metabolic rate and ultimately fitness‐related metrics like growth or the ability to migrate to their spawning ground (Bonneaud, Wilson, and Seebacher 2016).

Investigating the role of disease in animal ecology can help to better understand biological systems and the interactions between the host and its environment. Migratory animals such as salmonids are excellent model species for investigating host–pathogen relationships because non‐lethal tissue biopsies can provide a synoptic assessment of the individual's pathobiome using high‐throughput genome sequencing or microfluidics qPCR. Recent progress in pairing these assessments with electronic tagging and tracking technologies has provided insight into the role of pathogens in the behaviour and fate of migrating fishes (Bass et al. 2019; Teffer and Miller 2019). Finer scale links between animal physiology and its performance in the wild is possible with accelerometers, which are sensors that measure activity at the finest possible scales, which has an inherent link to the metabolism and individual performance (Gleiss, Wilson, and Shepard 2011; Lennox et al. 2023). We were particularly interested in the spawning migration of brown trout ( *Salmo trutta* ) when they would have been most exposed to marine pathogens that might affect the migration. Knowledge about the impacts of pathogens on trout is lacking and important given that open net‐pen fish farming in fjords throughout Norway is influencing pathogen exposure of trout during the marine sojourn (Vollset et al. 2021). We hypothesised that the pathobiome of migratory fish will have an effect on the energy expenditure during migration measured using accelerometer transmitters, that is, that elevated pathogen levels will have a deviating and consequently suboptimal activity pattern during the important final stage of in‐river migration. We tested this hypothesis using sea‐run brown trout in three rivers with different habitat and putative exposure pathogens.

## 2 Methods

### 2.1 Study Sites

**FIGURE 1:**
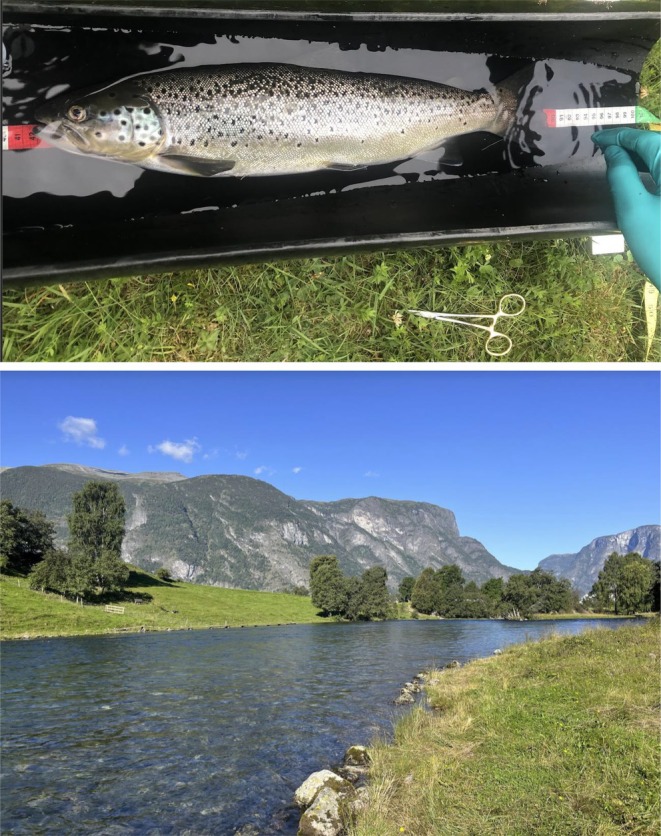
Sea‐run brown trout were captured and sampled in three Norwegian rivers, Vosso, Aurland (pictured) and Stjordal. Fish were tagged with acoustic accelerometer transmitters and biopsied for gill tissue to measure host gene expression and pathogens.

**FIGURE 2:**
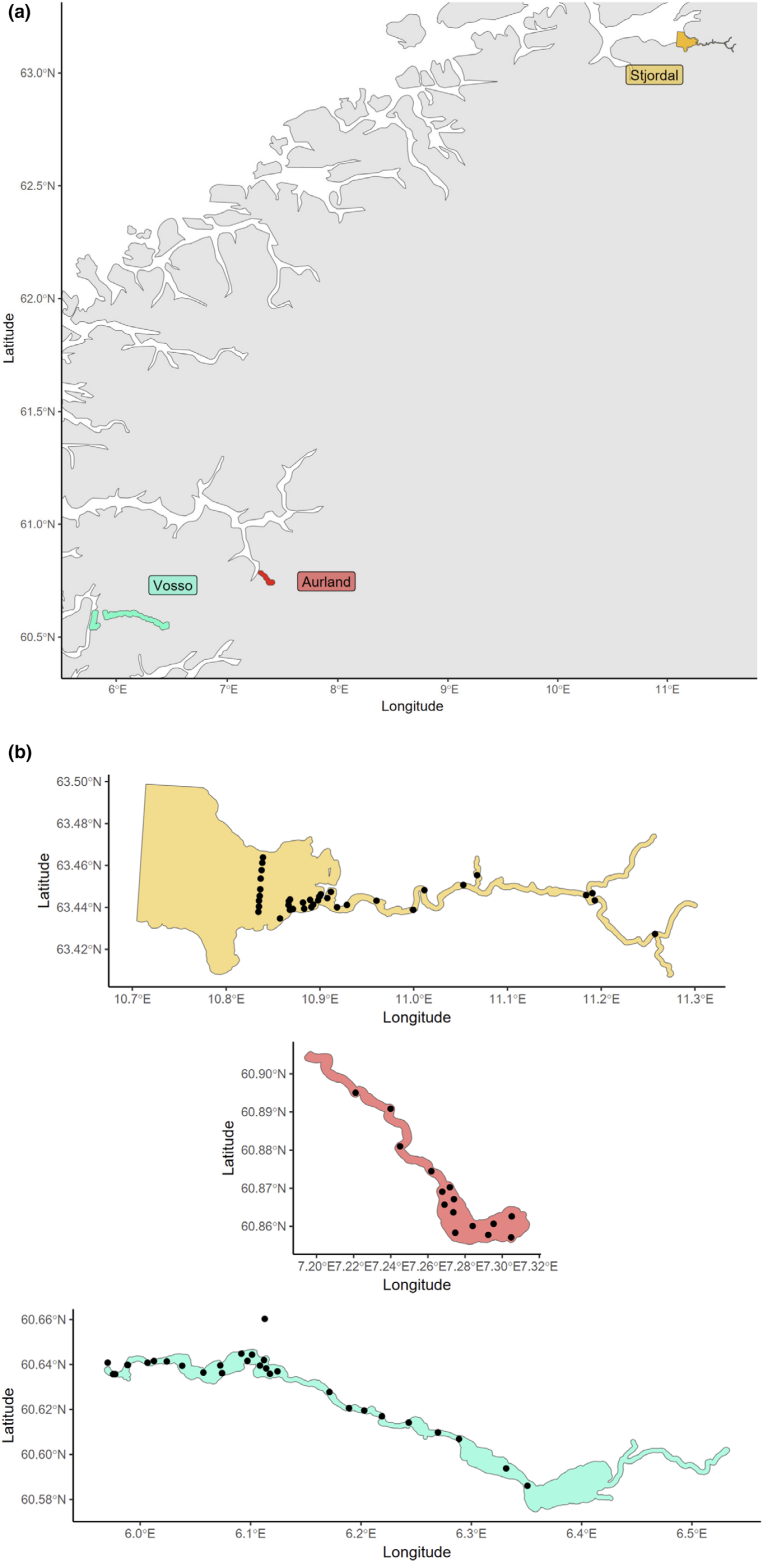
Map of Norway with three rivers that were sampled for this project. Vosso River is part of the Osterfjord, Aurland is a cold river in the Sognefjord and Stjørdal is river in the distal end of the Trondheimsfjord (a). In each river, we deployed acoustic receivers to detect transmissions from the tags recording the animal's root mean square acceleration (b).

Sea‐run brown trout (Figure 1) were captured, biopsied and tagged in three river‐fjord systems in different regions along the Atlantic coast of Norway in 2021, Vosso, Aurland and Stjørdal (Figure 2). Fish were captured during summer to gather a sample of fish that had been exposed to the marine environment for feeding and was preparing to return to the river to spawn. Presumably all fish (given the size distribution) had been to sea multiple times and some were likely repeat spawners, although this was not confirmed.

#### 2.1.1 Vosso River

Vosso is a large river system in western Norway that drains into Bolstadfjorden, a branch of the National Salmon Fjord Veafjorden, which is part of the fjord system Osterfjord surrounding the island Osterøy. Vosso is a National Salmon River and has two large lakes and an accessible stretch of about 35 km for anadromous fishes until a natural migration barrier in the uppermost river (Strandaelva). The lower reaches of Vosso are impacted by the water discharged from the Evanger power station, which abstracts hypolimnetic water from mountain lakes to Evanger Lake. Brown trout in Vosso migrate between the river and the fjord. Trout can move > 100 km away from the river but generally stay within the fjord during their sea sojourn either in the estuary or migrate to the more marine outer archipelago (Lennox et al. 2024). The marine habitat of sea‐run trout overlaps with open net‐pen fish farms mostly farming rainbow trout ( *Oncorhynchus mykiss* ) in Sørfjorden and Osterfjorden (migration distance from the river to Sørfjorden is approx. 35 km) but also salmon farms in the outer region of the fjord. Although Vosso is the main spawning area for trout in the fjords, several other nearby rivers (e.g., Dale, Ekso, Modalen and Romarheim) also host trout populations and there is a unique population of trout that lives and spawns in Bolstadfjorden, where a tidal stream creates suitable spawning conditions in the brackish water (Gabrielsen et al. 2021).

Trout (*N* = 20, 55 ± 14 cm SD TL) were captured in Bolstadfjorden in 2021 predominantly by kilenot (aka bag net) but also by fyke net. Individuals were tagged with Thelma LP13‐AT transmitters that alternatingly measured temperature and acceleration. Acceleration was measured at 12.5 Hz in a 27‐s sampling window. Receivers were placed in the fjord, river and lake sections of the system to cover key areas where the trout migrate in the system (Figure 2).

#### 2.1.2 Aurland River

Aurland is a small river with a deep lake, which drains into the Sognefjord in western Norway. Aurland has long been a popular destination for brown trout fishing and Atlantic salmon are contemporarily very rare in the system. The river is highly regulated by multiple hydropower stations and there is seasonal drawdown of the water from September–May. Aurland is naturally a very cold river system that is further cooled by releases of hypolimnetic water from the mountains into the lake Vassbygdivatnet. The marine habitat also overlaps with fish farming activity in the outer part of the Sognefjord, albeit the distance to these locations is more than 100 km because the inner part of the fjord is defined as a National Salmon Fjord.

Trout (*N* = 22, 53 ± 89 cm SD TL) were captured by recreational anglers fishing in the river with artificial flies exclusively. Individuals were tagged with Thelma LP13‐ADT transmitters measuring temperature, depth and acceleration. Acceleration was measured at 12.5 Hz in a 27‐s sampling window. Receivers were placed only in the river and lake sections of the watercourse and not in the fjord.

#### 2.1.3 Stjørdal River

Stjørdalselva is a large river that drains into inner parts of the Trondheimsfjord, Central Norway. The river discharge is influenced by hydro power production and has an average discharge of 78.44 m^3^/s. River Stjørdalselva and the Trondheimsfjord are categorised as a National salmon River—and fjord, which aims to ensure protection against new anthropogenic activities that can further deteriorate the living conditions for its salmon stock. The Trondheimsfjord basin is therefore free of open cage salmon farming, however, high‐intensity salmon farming is located just outside the fjord with a minimum distance in water of about 85 km from the river mouth. Several large and small salmon‐ and brown trout rivers drain out in the Trondheimsfjord.

The sea‐run brown trout included in this study (*N* = 24, 43 ± 75 cm SD TL) were captured in the estuarine parts of the river using rod and lures. Individuals were tagged with Innovasea V13‐A transmitters that measured acceleration at 12.5 Hz within a 27‐s sampling window. Receivers in Stjørdal were placed throughout the river and in the Trondheimsfjord, but were removed during wintertime.

### 2.2 Sampling

For this study, accelerometer‐derived activity metrics were linked to individual pathogen and gene expression of brown trout; therefore, we required data on the individual status at capture and activity during migration following sampling. To do so, trout from all three rivers were tagged with acoustic transmitters that included tri‐axial accelerometers and simultaneously they were biopsied for gill tissue from the anterior gill filaments. Gill tissue samples in RNAlater were refrigerated for 24 h before being transferred to a −18° freezer. Samples were shipped on dry ice to the Pacific Biological Station (Nanaimo, British Columbia, Canada) after several months frozen for molecular analysis.

#### 2.2.1 Molecular Analysis

To extract total RNA from gill tissue, the gill tissue was first homogenised in TRI reagent (produced by Ambion Inc.) and then underwent phase separation using 1‐bromo‐3‐chloropropane. The supernatants obtained were further processed to isolate pure total RNA using the Magmax‐96 for Microarrays RNA Kit (Ambion Inc.) on an automated Biomek NXP liquid handler (Beckman‐coulter), following the provided ‘spin method’. For reverse transcription, 400 ng of the extracted RNA was converted to cDNA using the SuperScript VILO Master Mix Kit (Invitrogen), adhering to the prescribed protocol.

High‐throughput qPCR was performed using the Fluidigm BioMark system (Standard Bio Tools, Markham, ON) to assess pathogen infection and stressor states detected in cDNA from gill tissues. This nanofluidic technology requires an initial specific target amplification (STA) for all assays, as described in Dhoubhadel et al. (2014), to account for the small size of reaction wells (7 nL), a process that was extensively evaluated for impacts on pathogen detections in Miller et al. (2016). For each assay, 1.3 μL of cDNA was pre‐amplified with 0.2 μM of primer pairs in a 5‐μL reaction volume using the TaqMan Preamp MasterMix (Applied Biosystems), with 14 amplification cycles. Post‐amplification, ExoSAP enzyme treatment (Affymetrix) was employed to eliminate unattached primers and the assays were diluted 1:5 in DNA Suspension Buffer (Teknova). To quantify pathogens, artificial positive constructs (APC) were synthesised, incorporating a sequence from each microbe assay region and an extra sequence for detecting vector contamination, as outlined in Miller et al. (2016). These APC clones were serially diluted and tested on the dynamic array to assess assay efficiency and determine microbe copy numbers. The BioMark Fluidigm Dynamic Arrays were operated as per the manufacturer's guidelines and Cycle threshold (Ct) values were recorded using the BioMark Real‐Time PCR analysis software (Standard Biotools).

Assays to viruses, bacteria and parasites known to infect salmon were run in duplicate along with a single housekeeping gene to assess sample quality on Fluidigm 96.96 Dynamic Arrays (Table 1). Pathogen assays were only considered positive when both duplicates showed detections; copy numbers were then averaged between replicates. On separate arrays, gene expression was examined for 63–82 curated biomarkers representative for thermal stress, hypoxia stress, salinity stress, smoltification and imminent mortality (Miller et al. 2017; Akbarzadeh et al. 2018; Houde, Günther, et al. 2019; Akbarzadeh et al. 2020; Akbarzadeh et al. 2021) as well as viral disease development (VDD) (Miller et al. 2017), immune stimulation and inflammation (Wang et al. 2023). For gene expression analysis, target cDNA amplicons for each gene were first enriched using the specific target amplification (STA) method as described above. All the amplified samples and the assays (primers and probes) were then run on four 96.96 gene expression dynamic arrays (Fluidigm Corporation, CA, USA) following the Fluidigm platform instructions as previously described (Akbarzadeh et al. 2018; Houde, Günther, et al. 2019; Akbarzadeh et al. 2020). Each gene expression chip also contained serial dilutions of a cDNA pool from either sea‐run brown trout or Atlantic salmon (1, 1/5, 1/25, 1/125 and 1/625) and a concentrated cDNA pool of samples from Atlantic salmon, Chinook, coho and/or sea‐run brown trout, as inter‐chip calibrator samples for normalisation of all salmon and trout species (including controls) run on the chips. These chips were processed and scored as outlined above and cycle threshold (Ct) values were recorded for each sample. PCR efficiency for each assay per chip was calculated using (10 ^1/slope^—1) × 100, where the slope was estimated by plotting the Ct over the serial dilutions of cDNA. Assay efficiencies ranged between 0.8 and 1.2 were considered as pass efficiencies. For determining the optimal normalisation gene(s), the expressions of all genes were evaluated across all the samples run on all chips using three independent software tools, that is, geNorm (https://genorm.cmgg.be), NormFinder (https://moma.dk/normfinder‐software) and a web‐based tool RefFinder (https://heartcure.com.au) to identify the most suitable gene pair with the best stability (lowest standard deviation). Next, the expression of the samples for each chip run were normalised in GenEx (ver. 7) to the geometric expression of two housekeeping genes, hk_MRPL40_113 and hk_CCDC84_114 (identified as the most suitable reference genes) with the ∆∆Ct method (Livak and Schmittgen 2001), using the calibrator sample (cDNA pool from all sea‐run brown trout salmon samples or other species for controls) to correct for any variance between dynamic arrays. Gene expression was then log transformed: log_2_ (2^−∆∆Ct^). The intersecting genes across species and years were then combined and the assays for two genes, ‘IM_H1F0_671’ and ‘OS_CFTR_I_206’, which were not amplified across all or some of the chips, were removed from further analysis. Once assays with poor efficiencies were removed, there remained 45 biomarkers across years and species that could be explored further (Table 2).

**TABLE 1:** Pathogens were measured from gill biopsies for sea‐run brown trout in Vosso, Aurland and Stjørdal rivers in Norway.

Class	Microbe	Assay Abbreviation
Bacteria	*Aeromonas salmonicida*	ae_sal
Bacteria	Candidatus Branchiomonas cysticola	c_b_cys
Bacteria	Candidatus syngnamydia salmonis (gill chlamydia)	sch
Bacteria	*Flavobacterium psychrophilum* (bacterial cold‐water disease‐BCWD)	fl_psy
Bacteria	*Moritella viscosa*	mo_vis
Bacteria	Piscichlamydia salmonis	pch_sal
Bacteria	*Piscirickettsia salmonis*	pisck_sal
Bacteria	*Renibacterium salmoninarum*	re_sal
Bacteria	Rickettsia‐like organism (Strawberry disease)	rlo
Bacteria	*Tenacibaculum dicentrarchi*	te_dic
Bacteria	Tenacibaculum finnmarkense	te_fin
Bacteria	*Tenacibaculum maritimum*	te_mar
Bacteria	*Vibrio anguillarum*	vi_ang
Bacteria	*Vibrio salmonicida*	vi_sal
Bacteria	*Yersinia ruckeri* (Enteric redmouth disease)	ye_ruc
Parasite	Ichthyobodo necator, Ichthyobodo salmonis	Ic_spp; Ich_costia
Parasite	Ichthyophonus hoferi	ic_hof
Parasite	Ichthyophthirius multifiliis	ic_mul
Parasite	Loma salmonae (Loma sp.)	lo_sal
Parasite	Neoparamoeba perurans	ne_per
Parasite	Paranucleospora theridion	pa_ther
Parasite	Parvicapsula pseudobranchicola	pa_pse
Parasite	Spironucleus salmonicida (Diplomonadida; Hexamitidae); aka Hexamita salmonis	sp_sal
Parasite	Tetracapsuloides bryosalmonae	te_bry
Virus	Atlantic salmon paramyxovirus (ASPV)	aspv
Virus	Cutthroat Trout Virus 2 (CTV2)	ctv‐2
Virus	Erythrocytic necrosis virus (ENV)	env
Virus	Infectious pancreatic necrosis virus (IPNV)	ipnv
Virus	Infectious salmon anaemia virus (ISAV)	isav
Virus	Pacific salmon nidovirus 1	psnv‐1
Virus	Piscine myocarditis virus (PMCV)	pmcv
Virus	Piscine orthoreovirus 1 (PRV1)	prv‐1
Virus	Piscine orthoreovirus 3 (PRV3)	prv‐3
Virus	Putative Rota Virus	p‐rotav
Virus	Putative Toti‐like virus	p‐totiv
Virus	Salmon alphavirus 1, 2, and 3 (SAV)	sav
Virus	Salmon Gill Pox Virus (SGPV)	sgpv
Virus	Viral encephalopathy and retinopathy virus (VER)	ver
Virus	Viral hemorrhagic septicemia virus (VHSV)	vhsv

*Note:* Pathogen loads were used to calculate relative infection burden of individual trout.

**TABLE 2:** Assays for 48 host genes (biomarkers) tested on sea‐run brown trout salmon over multiple years, categorised based on original stressor panels.

Gene name	Fit‐chips name	Primers/Probes
Housekeeping (HK)		
Coiled‐coil domain‐containing protein 84	HK_CCDC84_114*^HK^	F‐GCTCATTTGAGGAGAAGGAGGATG R‐CTGGCGATGCTGTTCCTGAG P‐TTATCAAGCAGCAAGCC
39S ribosomal protein L40, mitochondrial precursor	HK_MRPL40_113*^HK^	F‐CCCAGTATGAGGCACCTGAAGG R‐GTTAATGCTGCCACCCTCTCAC P‐ACAACAACATCACCA
S100 calcium‐binding protein	HK_S100_10	F‐GTCAAGACTGGAGGCTCAGAG R‐GATCAAGCCCCAGAAGTGTTTG PAAGGTGATTCCCTCGCCGTCCGA
Thermal stress (TM)		
FK506‐binding protein 10 precursor (chr.19)	TM_FKBP10_4_583*^TM^	F‐CCTGAAGAGATCATTGCTGACATG R‐GACGATGACCCCATCCTTGT P‐TCAGGAACCAGGACCG
Heat shock protein 90 alpha	TM_HSP90AA_269*^TM^	F‐ATGACCCTCAGACACACTCCAA R‐CCTCATCAATACCCAGTCCTAGCT P‐CGCATCTACAGAATGA
Heat shock protein 90 alpha like	TM_HSP90a_271*^TM^	F‐TTGGATGACCCTCAGACACACT R‐CGTCAATACCCAGGCCTAGCT P‐CCGAATCTACCGGATGAT
Mitogen‐activated protein kinase 14	TM_MAP3K14_308*^TM^	F‐GCTCCCTGGGTTCATGGAT R‐GCCTCCCTTCAGCAGAGACA P‐CCAGCAATAGCTTATG
Serpin H1 precursor (HSP47) (chr.20)	TM_SERPIN_20_379*^TM^	F‐ACTATGACCACTCGAAGATCAACCT R‐CCCATTCGTTGATGGAGTTCA P‐AGGGACAAGAGGAGC
Serpin H1 precursor (HSP47) (chr.9)	TM_SERPIN_9_380*^TM^	F‐GAGGTCAGCGACCCAAAGAC R‐GCCGTAGAGGCGGTTACTGAT P‐CGGAACGTCACATGGA
Imminent mortality (IM)		
Arrestin domain containing 2	IM_ARRDC2_663*^IM^	F‐AAGAAAGCCAAGGCGTGAGTAA R‐TCGGTTGCCAGGGTTAGC P‐TGGAGGACAAATCGGA
C‐type lectin domain family 4, member E	IM_CLEC4E_666*^IM^	F‐CCTGAGGGCTGGATTCATGT R‐TCGGCCAGTCCATCTTGTC P‐TGAGAAATGTTACTCCTTCAGT
Glutamate‐ammonia ligase (glutamine synthetase)	IM_GLUL_670*^IM^	F‐GTTCCAGGTTGGCCCTTGT R‐CCTAGCTGCCCAAAGGTGATC P‐AAGGCATCAGCATGGG
Nuclear protein 1	IM_NUPR1_677*^IM^	F‐GGAAGCCAGCGACAATACCA R‐GGGTTAGCCGTCCGATTTG P‐CACGAGCGCAAGCT
Ornithine decarboxylase 1	IM_ODC1_678*^IM^	F‐CCAGAAGGCTCCCTGTTTCA R‐GCAGCCATTTCCTGGAGAAG P‐ACAACCCAATCTCA
Transgelin 3	IM_TAGLN3_681*^IM^	F‐TGGCTCAAGGACGGATGTG R‐GGATCTTCCTGATGGGCTTGT P‐TGTGTGAACTGATCAACAG
Mortality‐related signature (MRS)		
ATP synthase lipid‐binding protein, mitochondrial precursor	MRS_ATP5G3_181	F‐GGAACGCCACCATGAGACA R‐CGCCATCCTGGGCTTTG P‐AGCCCCATTGCCTC
NKA β1	MRS_NKAB1_328	F‐CGTCAAGCTGAACAGGATCGT R‐CCTCAGGGATGCTTTCATTGGA P‐CCTTGGCCTGAAGTTG
Smoltification/osmoregulation (OS)		
C‐C motif chemokine 19	OS_CCL19_578	F‐ACCTGGGTTACAGACCTGATGAA R‐TGGTTTCGTGGCATTTCTTG P‐CTCATGGACCGCCTCA
CC chemokine 4	OS_CCL4_195	F‐TCTCTTCATTGCAACAATCTGCTT R‐ACAGCAGTCCACGGGTACCT P‐CTACGCAGCAGCATT
CCAAT/enhancer binding protein (C/EBP), beta	OS_CEBPB_665*^IM^	F‐AACTGGCCGCAGAGAATGAC R‐AAGTTACGCAGAGTGGCAAGCT P‐TTTACAAAAACGCGTGGAGC
Haemoglobin subunit α	OS_HBA_254*^IM^	F‐GCCCTGGCTGACAAATACAGA R‐GAGCAGGAACTGGAGTCCAATG P‐ACCATCATGAAAGTCC
Na/K ATPase α‐1a (freshwater)	OS_NAKATPASE1a_330	F‐TGGAATCAAGGTTATCATGGTCACT R‐CCCACACCCTTGGCAATG P‐ATCATCCCATCACTGCGA
Na/K ATPase α‐1b (saltwater)	OS_NAKATPASE1b_333	F‐GCCTGGTGAAGAATCTTGAAGCT R‐GAGTCAGGGTTCCGGTCTTG P‐CCTCCACCATTTGCTCA
Regulation of G protein signalling 21	OS_RGS21_597	F‐TCCCGACTACAGCGCAGAT R‐TCCTCAGGGCTAAGTCGTTCA P‐TTCCCAATCCCCC
Chloride levels (CL)		
Histocompatibility 2, class II antigen E beta	CL_H2EB1_672	F‐CAGTTGAGCCCCATGTCAGA R‐TCAGCATGGCAGGGTGTCT P‐TGAGCTCAGTGACTCC
HLA class 2 gamma	CL_HLA21‐G1_684	F‐CCAGGACGTTATCCTCCCAAT R‐GAGAAGACACGCCAGCACTGT P‐AGGGCCTCTAACAGC
Invariant chain‐like protein 2	CL_ICLP2_674	F‐CAGCAGAAGGGTCCAACAAGAG R‐TCCTGCAGGTCTTTAATGTCGTT P‐TTCAAGATAGCTGGTTTCAC
Viral disease diagnostic (VDD)		
Probable E3 ubiquitin‐protein ligase HERC6	VDD_HERC6_77*^VDD^	F‐AGGGACAACTTGGTAGACAGAAGAA R‐TGACGCACACACAGCTACAGAGT P‐CAGTGGTCTCTGTGGCT
Interferon‐induced protein with tetratricopeptide repeats 5	VDD_IFIT5_2_83*^VDD^	F‐CCGTCAATGAGTCCCTACACATT R‐CACAGGCCAATTTGGTGATG P‐CTGTCTCCAAACTCCCA
IFN‐induced protein 44–1	VDD_IFI44A_81*^VDD^	F‐CGGAGTCCAGAGCAGCCTACT R‐ CCAGTGGTCTCCCCATCTC P‐CGCTGGTCCTGTGTGA
MX gene	VDD_MX_86*^VDD^	F‐AGATGATGCTGCACCTCAAGTC R‐CTGCAGCTGGGAAGCAAAC P‐ATTCCCATGGTGATCCGCTACCTGG
Zinc finger NFX1‐type	VDD_NFX_87*^VDD^	F‐CCACTTGCCAGAGCATGGT R‐CGTAACTGCCCAGAGTGCAAT P‐TGCTCCACCGATCG
VHSV‐inducible protein‐4	VDD_VHSV4_107	F‐CTCTCGTAAAGCCCCACATC R‐GGCGACTGCTCTCTGATCT P‐AAACTGCACGTCGCGC
*Oncorhynchus mykiss* VHSV‐induced protein‐10	VDD_VHSV_108	F‐GCAAACTGAGAAAACCATCAAGAA R‐CCGTCAGCTCCCTCTGCAT P‐TGTGGAGAAGTTGCAGGC
Inflammation (IF)		
ES1 protein homologue	IF_ES1_668*^IF^	F‐CGGCAACTTCCATGAAGGA R‐GGACCTCCCCCACTTTCTTATT P‐TGGGCTGTAAACACG
Interleukin 11	IF_IL11_291*^IF^	F‐GCAATCTCTTGCCTCCACTC R‐TTGTCACGTGCTCCAGTTTC P‐TCGCGGAGTGTGAAAGGCAGA
Matrix metalloproteinase 13	IF_MMP13_315*^IM^*^IF^	F‐GCCAGCGGAGCAGGAA R‐AGTCACCTGGAGGCCAAAGA P‐TCAGCGAGATGCAAAG
Matrix metalloproteinase 25 precursor	IF_MMP25_316*^IS^	F‐TGCAGTCTTTTCCCCTTGGAT R‐TCCACATGTACCCACACCTACAC P‐AGGATTGGCTGGAAGGT
Thioredoxin	IF_TXN_683*^IF^	F‐CAAGAATGTGGTTTTCCTCAAGGT R‐GCATTTGATGTCACAGTGTTTGG P‐TGGACGAGGCAGCG
Immune stimulation (IS)		
Beta 2‐microglobulin (ClassI MHC)	IS_B2M_182* ^IS^	F‐TTTACAGCGCGGTGGAGTC R‐TGCCAGGGTTACGGCTGTAC P‐AAAGAATCTCCCCCCAAGGTGCAGG
C5a receptor	IS_C5aR_577*^IS^	F‐ACGCACCTTGAGGGTCATT R‐CAGTGGAAACCAGCACAGG P‐TTGCCGTGTCGCTGAGCTTCTT
Cluster of differentiation 83	IS_CD83_579*^IS^	F‐GTGGCGGCATTGCTGATATT R‐CTTGTGGATACTTCTTACTCCTTTGCA P‐CACCATCAGCTATGTCATCC
Interferon α	IS_IFNa_282*^IS^	F‐CGTCATCTGCAAAGATTGGA R‐GGGCGTAGCTTCTGAAATGA P‐TGCAGCACAGATGTACTGATCATCCA
Interleukin 1b	IS_IL1B_295*^IS^*^IF^	F‐AGGACAAGGACCTGCTCAACT R‐CCGACTCCAACTCCAACACTA P‐TTGCTGGAGAGTGCTGTGGAAGAA
Major histocompatibility complex I	IS_MHC1_313*^IS^	F‐GCGACAGGTTTCTACCCCAGT R‐TGTCAGGTGGGAGCTTTTCTG P‐TGGTGTCCTGGCAGAAAGACGG
Retinoic acid inducible gene 1	IS_RIG1_361*^IS^	F‐ACAGCTGTTACACAGACGACATCA R‐TTTAGGGTGAGGTTCTGTCCGA P‐TCGTGTTGGACCCCACTCTGTTCTCTC
Serum amyloid protein a	IS_SAA_370*^IS^	F‐GGGAGATGATTCAGGGTTCCA R‐TTACGTCCCCAGTGGTTAGC P‐TCGAGGACACGAGGACTCAGCA

*Note:* Mortality‐related signature (MRS) is a curated panel discovered in association with migratory losses in salmon (Miller et al. 2011) and contains genes from multiple physiological pathways. Viral disease development (VDD) is a curated panel that when co‐expressed indicates a viral disease state across multiple RNA viruses infecting salmon (Miller et al. 2017). Asterisks followed by pathway abbreviation signify which genes were used for each biological pathway panel.

The host transcription panels included in this study were developed and validated previously in *Oncorhynchus* spp. to recognise specific stressor (thermal, osmotic, hypoxia, imminent mortality; Houde, Günther, et al. 2019; Akbarzadeh et al. 2020) and disease (Miller et al. 2017) states on ‘Salmon Fit‐Chips’. These panels generally contain genes that are co‐activated under specific stressor states, although constitutively downregulated genes are occasionally included. For Pacific salmon species, random forest classifiers to recognise the presence of each stressor/disease state have been developed based on control samples from multi‐stressor challenge studies, with probability thresholds established that maximise sensitivity and specificity (e.g., Akbarzadeh et al. 2024). In general, there is strong overlap across species in biomarker panels predictive of stressor and disease states (e.g., Chinook: Houde, Akbarzadeh, et al. 2019, Coho: Akbarzadeh et al. 2024), providing support for their application in salmonids for which validation studies have not yet been undertaken (e.g., Lennox et al. 2020). In this study, we applied Salmon Fit‐Chips ‘off‐species’, using the biomarkers that provide the most consistent signals associated with each physiological stressor/disease state across five salmon species and incorporated physiological control samples from challenge studies in other salmonid species.

#### 2.2.2 Accelerometry

Individual performance of sea‐run brown trout was measured by root mean square (RMS) acceleration recorded on tri‐axial accelerometers in Innovasea and Thelma Biotel acoustic transmitters. To gather these data, trout from the three river systems were tagged with internally implanted acoustic transmitters. In all cases, trout were anaesthetised using either Aqui‐S (Vosso, Aurland) or Benzoak Vet (Stjørdal) and the tag was inserted by making a small incision with a sterile scalpel, inserting the transmitter and suturing the wound closed using 4/0 sutures. All surgeries were conducted according to approval by the Norwegian Food Safety Authority (Mattilsynet). Following a brief recovery, the fish regained equilibrium and startle responses and were released back to the river to continue the migration. The accelerometers inside the tags measured individual acceleration using a tri‐axial accelerometer set to sample acceleration on all three axes at 12.5 Hz within a 27‐s sampling window, which was converted to a measure of RMS acceleration and sent with the tag ID, which was then recorded by receivers in each of the three systems (Figure 2). Acceleration measurements were delivered with every transmission of the Innovasea transmitters (nominal delay = 90 s), every second transmission for the Thelma LP13‐AT transmitters (nominal delay = 180 s) and every third transmission for the Thelma LP13‐ADT (nominal delay = 270 s). For all models, raw transmitted values were converted to acceleration by a standard equation provided by the manufacturers.

#### 2.2.3 Linking Energy Use and Swimming Performance

The link between acceleration readings and energy consumption was explored by running respirometry trials on six wild‐caught sea‐run brown trout from River Stjørdalselva, tagged with Innovasea V13‐A acceleration sensors, in a 90‐L Loligo swim chamber. After capturing and tagging in river Stjørdalselva, the fish were transported in a 370 L holding tank to NTNU Sealab and transferred to 2 m diameter holding tanks for at least 7 days acclimatisation to lab conditions. Following acclimatisation, the fish were transferred to the swim chamber for a 24‐h restitution period at flows ~0.5 bl/s prior to swim trials. The swim trial was performed by increasing the waterflow with ~0.5 bl/s every 20 min and measuring oxygen consumption and the trial ended when the fish was exhausted and ceased normal swimming behaviour. During the swim challenge, acceleration recordings by the tags were logged by a Innovasea VR2W receiver mounted in the water reservoir of the Loligo swim chamber. Oxygen measurements in the chamber were measured and related to swimming performance using a regression model.

### 2.3 Data Analysis

Prior to analysis of data from the free swimming brown trout, acceleration profiles for all fish were visualised and dead fish were identified by sudden cessation of acceleration (i.e., zero values). Mortality timestamps were extracted and data series were truncated for fish that died, so that models were not influenced by overrepresentation of zero values from dead fish. There was uncertainty as to whether some transmitters had malfunctioning accelerometer sensors that never properly measured acceleration, so not all fish that were removed were necessarily deceased. All data analyses were performed in R software (R Core Team 2024).

#### 2.3.1 Pathogen Profiles

Pathogen assays with detections in both of the duplicated samples were averaged and singletons treated as null detections. For each assay, averaged detections Cts below known 75% confidence intervals for limit of detection were set to zero (Miller et al. 2016). Relative infection burden (RIB, Bass et al. 2019) was subsequently calculated by dividing log transformed copy numbers of each pathogen detection to the highest recorded log transformed pathogen reading of each pathogen species from a sample population including a larger group of sea‐run brown trout samples from four Norwegian river systems. Pathogen copy numbers were fitted to a non‐metric multidimensional scaling (NMDS) analysis using the *metaMDS* function in R vegan (Oksanen et al. 2022). Pathogens with zero detections were removed from NMDS because they could not be fit to the analysis, leaving 14 pathogens. Fifteen trout without any pathogens detected were also excluded, seven from Aurland, six from Vosso and two from Stjordal. The final number of trout used for the model was then 66.

As a statistical test for differences among the three rivers, permutated multivariate analysis of variance (perMANOVA) was performed, using the adonis2 function in R vegan and 999 permutations. Visualisation with ggplot2 (Wickham 2016) included a 50% ellipsoid polygon around each river. An indicator species analysis was run using the *multipatt* function in the indicspecies package to evaluate whether distinct pathogens were associated with specific rivers (De Cáceres and Legendre 2009).

#### 2.3.2 Transcription Panels

To evaluate stressor signals from gene panels, we ordinated the raw gene expression data along with control samples for each gene panel using a principal components analysis with the *rda* function in the R package vegan (Oksanen et al. 2022). The first two PC axes were visualised for each gene panel and assessed for established patterns of gene expression associated with individual stressor/disease states. By overlaying positive and negative control samples from challenge studies, we ensured that stressor states were properly differentiated within our sample. Sea‐run brown trout that clustered with positive control samples were considered ‘stressed’ under each panel; instead of applying cut‐off thresholds, the loading along the PC axis that was most consistent with the stressor profile was extracted and used as a covariate in our models. Only two panels, thermal stress (PC1) and viral disease development (PC2), showed patterns consistent with their presence in sea‐run brown trout samples. The PC1 axis of the PCA for thermal stress (*p* = 0.04) and the PC2 axis of the PCA for viral disease development (*p* = 0.94) were then also layered into the non‐metric multidimensional scaling plot (see above) using the *envfit* function in the vegan package to illustrate relationships between these biomarker panels and pathogen profiles of the trout from the three rivers.

#### 2.3.3 Proximate Effects of Pathogen Infection on Trout Acceleration

Transmitted acceleration data sets from receivers deployed in Aurland, Stjørdal, and Vosso were combined. To reduce temporal autocorrelation, the RMS acceleration data were subset to use only the first non‐zero data RMS acceleration measurement per hour for each fish. The dataset for modelling included individual ID, river of origin, days after release, hour of day, length of the fish, the relative infection burden calculated from the copy numbers of pathogen gene expression (per Bass et al. 2019), the principal components analysis axis associated with thermal stress, and the principal components axis associated with viral disease development. RMS acceleration data were modelled by hierarchical generalised additive modelling (Pedersen et al. 2019) using the *bam* function in mgcv (Wood 2017). Hour of day was considered as a cubic circular smoother (i.e., bs=“cc”) with a smooth effect having *K* = 5, days after release also had a smooth effect with *K* = 5. River of origin, total length and relative infection burden were fixed effects and individual ID was fitted with a random intercept (i.e., bs=“re”). The model was fitted with fast restricted maximum likelihood (i.e., method=“fREML”) and with a Gamma family and log link function (i.e., family = Gamma(link=“log”)). The model was fitted a second time using the copy numbers for *Candidatus* B. cysticola instead of relative infection burden to test whether loads of this specific pathogen, being most abundant across the sample, similarly affected acceleration.

For linking swimming acceleration recorded in the wild to energy use, a generalised additive model was fitted to the oxygen consumption readings recorded by the Loligo swim chamber and recorded sensor readings using the bam function with fREML, Gamma family and log link function. Here, acceleration was considered as a smooth effect with *K* = 5 and transmitter ID as a random effect to account for repeated measures and individual variation in respiration.

## 3 Results

### 3.1 Pathogen Profiles

**FIGURE 3:**
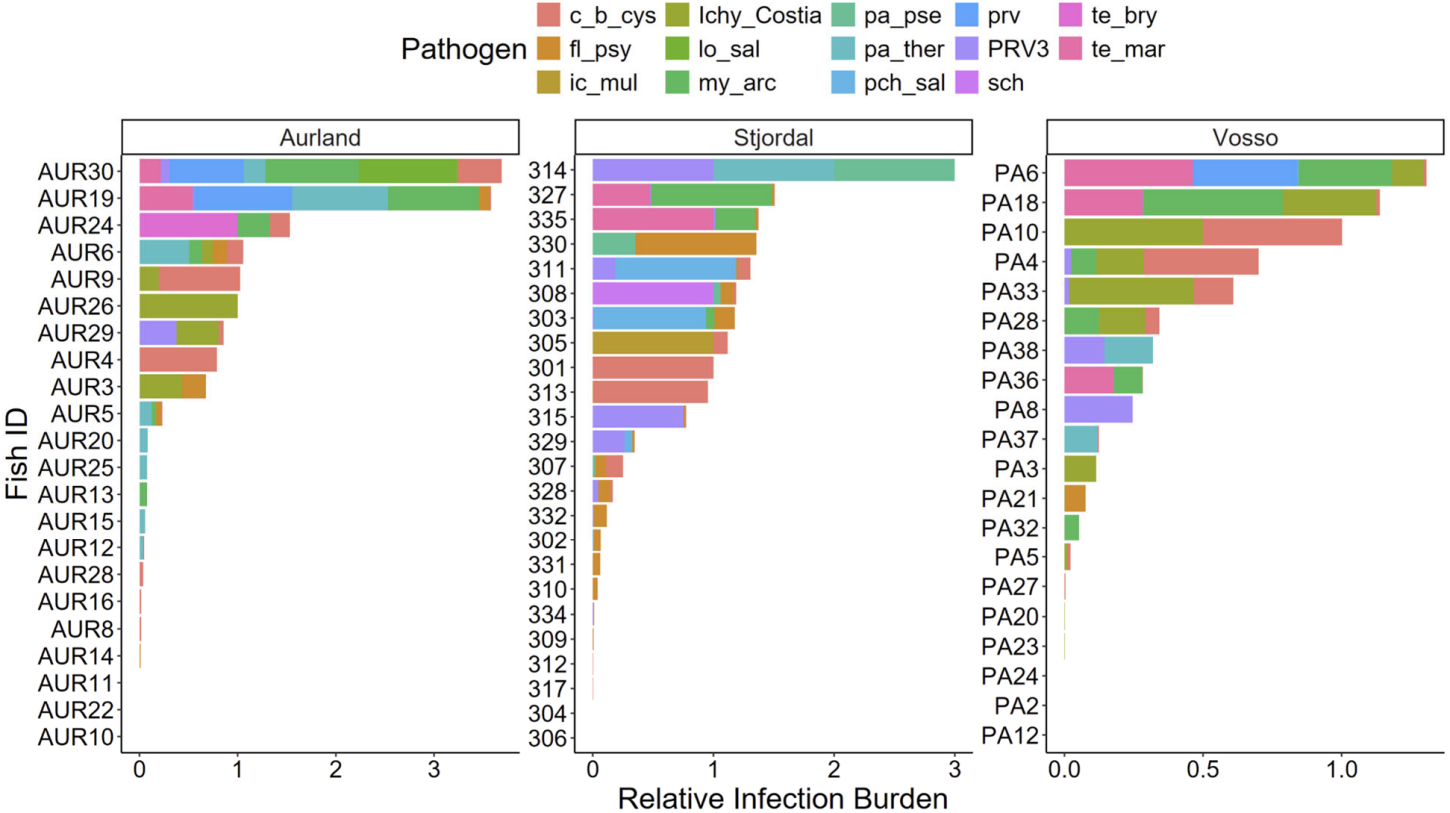
Relative infection burden (RIB) of sampled fish from River Aurland, River Stjørdal and River Vosso. Colours indicate the influence of specific pathogens on the individual's overall RIB. Identifying fish numbers indicate the individual that a sample was drawn from. Pathogen codes can be referenced in Table 1.

**FIGURE 4:**
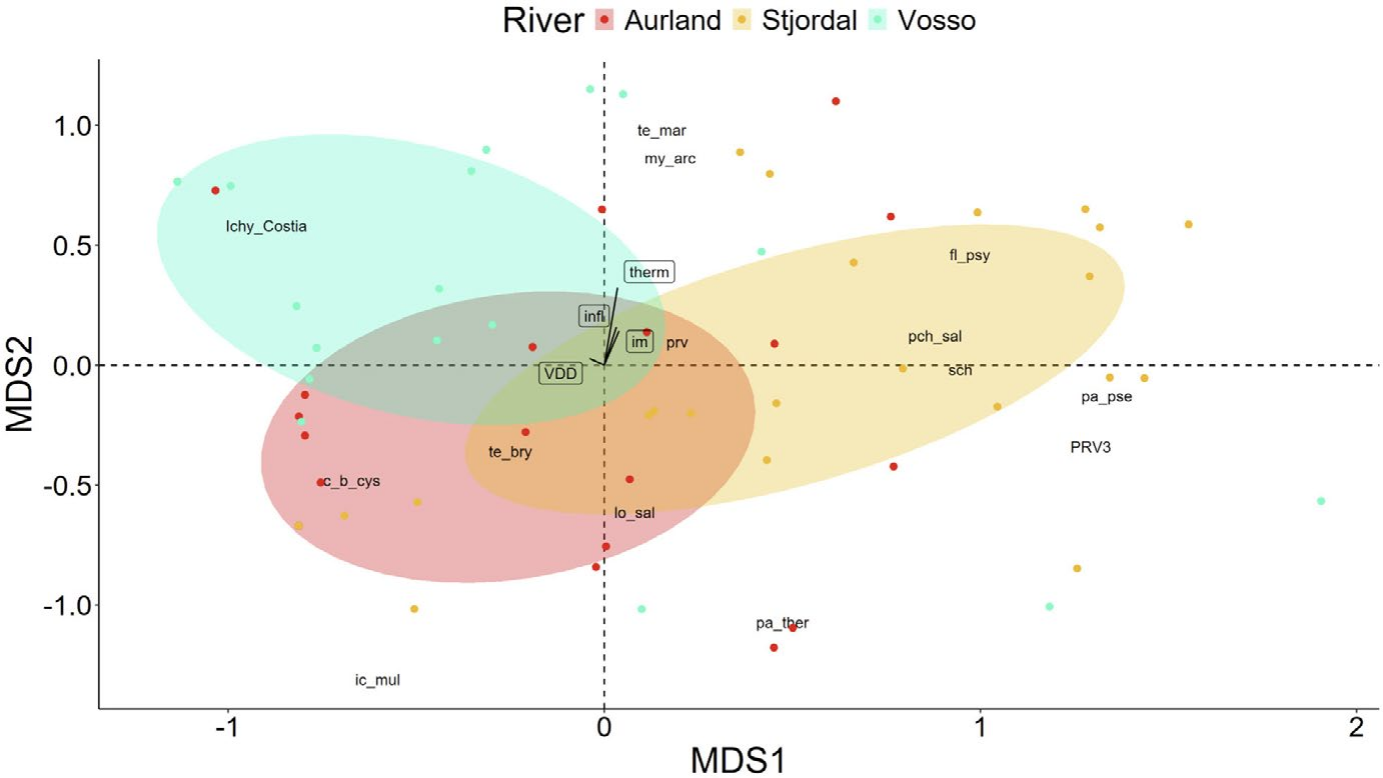
Non‐metric multidimensional scaling of pathogens (log copy number) and returning sea‐run brown trout to three Norwegian rivers, Aurland, Stjørdal and Vosso. Ellipsoid polygons shade 50% of the area for each river, indicating levels of separation in the pathogen communities. Reference species codes in Table 1.

The three individuals in the data set with the highest relative infection burden were all from Aurland, and on average Aurland trout had the highest values (mean = 1.50 ± 1.51 SD) followed by Stjørdal (mean = 1.18 ± 0.81 SD) and Vosso (mean = 1.06 ± 0.94 SD; Figure 3). Sea‐run brown trout in the three rivers had different pathogen profiles, provided by ordination using NMDS (Figure 4). perMANOVA suggested significantly different pathogen composition among the three rivers (*F* = 3.21, *p* < 0.01). An indicator species analysis suggested that *Flavobacterium psychrophilum* and *Piscichlamydia salmonis* were indicators for Stjørdal and that *Ichthyobodo costia* was an indicator for Vosso and Aurland.

### 3.2 Transcription Panels

According to the control plots using principal components analysis, thermal stress ordinated along PC1 and viral disease development along PC2 (data not shown). There was weak evidence of a relationship between thermal stress‐related gene expression and pathogen profiles based on the envfit (*p* = 0.04).

### 3.3 Short‐Term Effects of Pathogen Infection on Trout Acceleration

**FIGURE 5:**
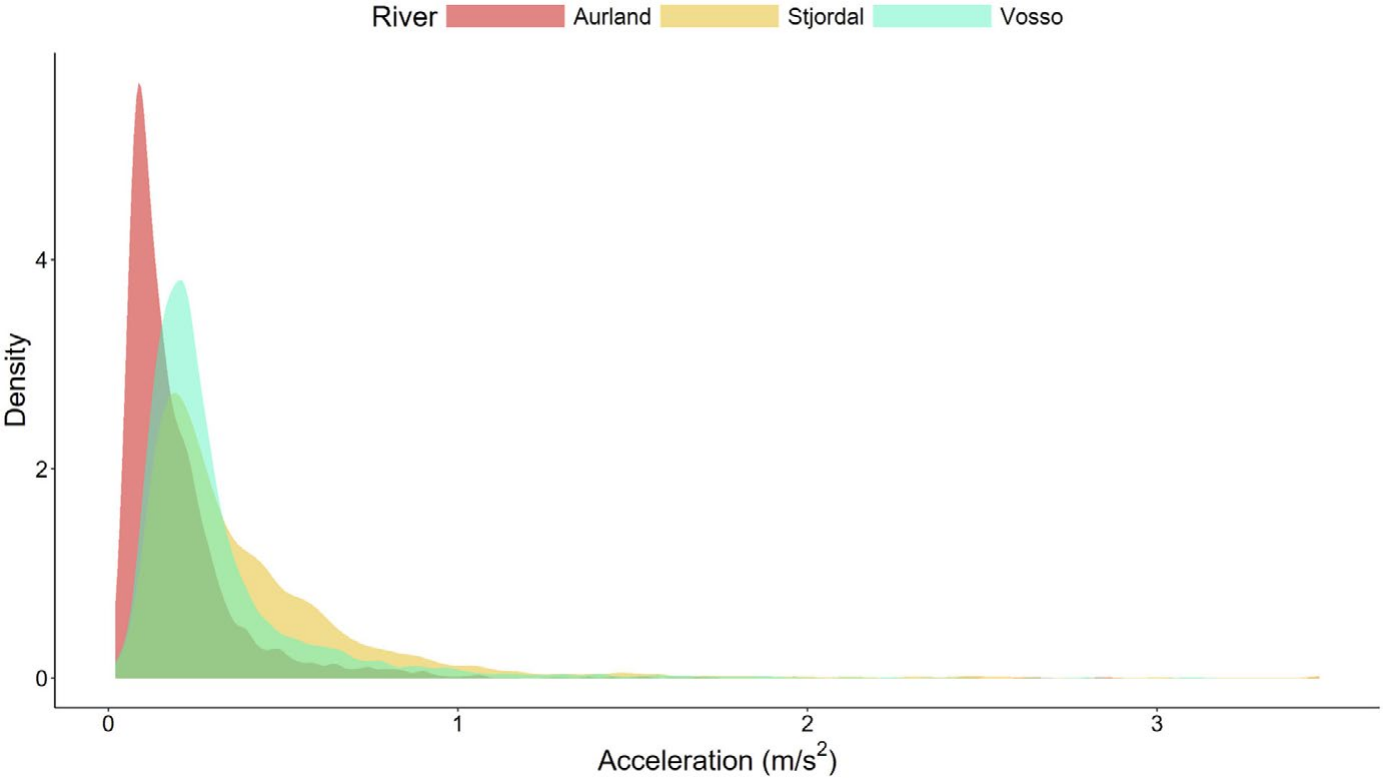
Density of root mean square acceleration values, measured at 12.5 Hz in three dimensions within a 27‐s sampling window, for brown trout in the river systems sampled during 2021. Data are subset to the first 14 days after release to match values to the pathogen profile sampled from gill biopsies taken at the time of capture.

**FIGURE 6:**
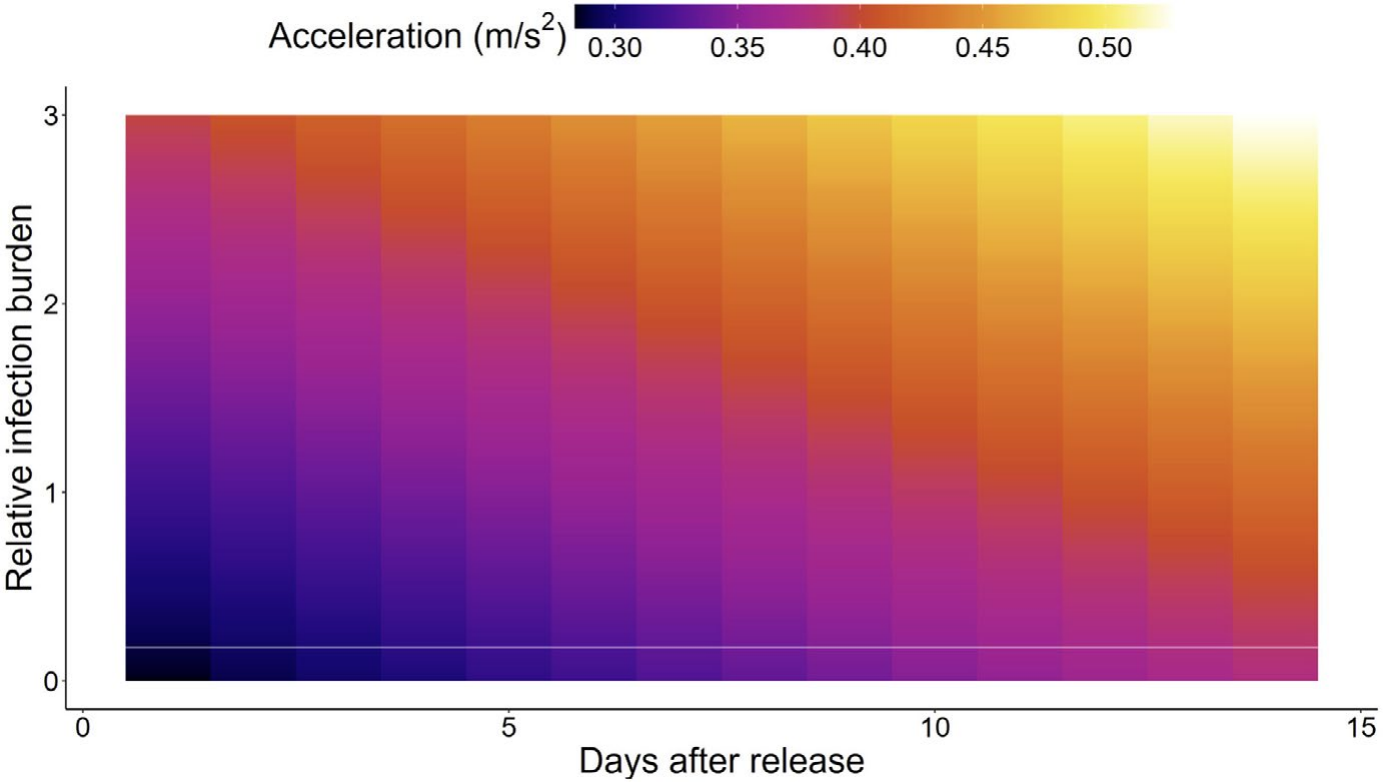
Predicted activity of sea‐run brown trout within 2 weeks of release. Gill biopsies analysed for pathogens and summarised as relative infection burden were fitted to a model including temporal variables and random effects. Here it is illustrated how model predictions reveal increasing activity with increasing days after release and higher relative infection burden from pathogens.

**FIGURE 7:**
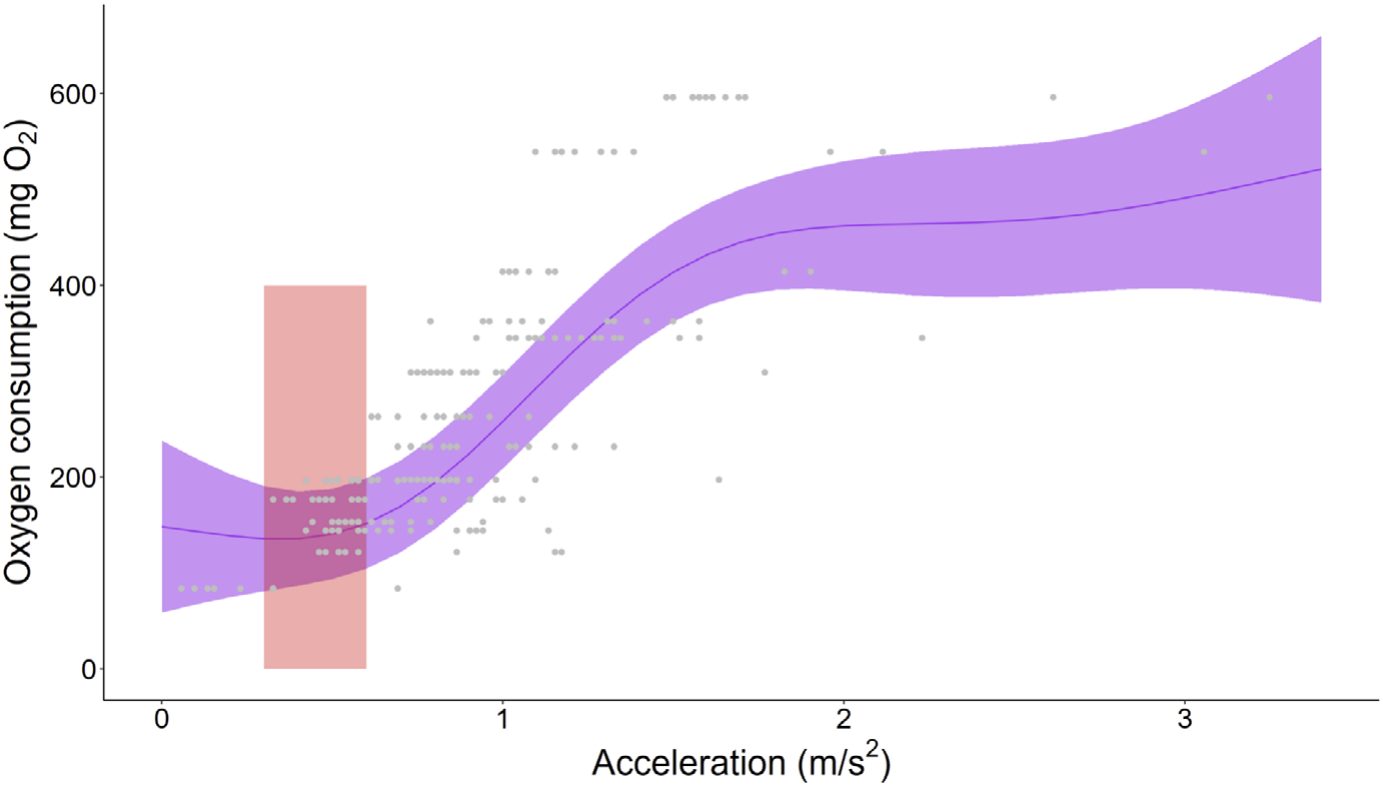
Modelled relationship between accelerometer transmitter derived acceleration, provided by the tag in root mean square of the three axes of acceleration and oxygen consumption in a swim tunnel respirometer. The ribbon provides model predictions from a generalised additive mixed model demonstrating a putative gait transition around an RMS acceleration of 1. The red polygon indicates the model predictions for acceleration of the trout in the field, indicating generally low levels of activity and near baseline metabolism in most contexts. Raw data that were used to parameterise the model are included as grey points.

Subsetting the data to the first value per fish per hour and the first 2 weeks provided 22,332 data points in the series from 74 wild trout in their natural environments in Vosso, Aurland and Stjørdal. There were significant effects of days after release (*F* = 33.87, *p* < 0.01), indicating that there were temporal impacts on the activity of the trout, although trout indicated no evidence of diel variation in activity (*F* = 3.82, *p* = 0.27). Longer trout tended to have less activity recorded by the tags (*t* = −3.01, *p* < 0.01) and there were also significant effects of the water courses such that trout in Aurland, with the highest relative infective burden at a population‐level, had lower activity than in Vosso (*t* = 4.53, *p* < 0.01) or Stjørdal (*t* = 5.45, *p* < 0.01; Figure 5). At an individual level, there was a significant effect of relative infection burden on acceleration such that relative infection burden resulted in increased activity (*t* = 2.34, *p* = 0.02; Figure 6). However, the change in acceleration attributable to increased relative infection burden had a relatively small effect size relative to the potential variation in activity measures (Figure 7). *Candidatus* B. cysticola was the most prevalent agent observed across the dataset, with copy numbers ranging from 0 to 10^5. Refitting the model using *Ca*. B. cysticola copy numbers, rather than relative infection burden, suggested that this bacterium itself was not sufficient to explain patterns in acceleration (*t* = 0.29, *p* = 0.77).

### 3.4 Respirometry

The respirometer data showed a sigmoidal relationship between oxygen consumption and acceleration in the transmitter tags, where oxygen consumption did not start increasing until the acceleration data increased beyond approximately 0.6 m/s^2^. In this phase, the oxygen consumption was ~200 mg O_2_/kg/h. As acceleration increased from 0.6 to 1.7 m/s^2^, the oxygen consumption increased rapidly, corresponding to ramping activity, eventually stabilising at about 400–550 mg O_2_/kg/h at high activity. To show how pathogen‐induced activity increase affected oxygen consumption, we overlaid the average effect of pathogens on the oxygen–activity relationship (approximately a change from 0.3 to 0.6 m/s^2^ at high RIB; Figure 7). This demonstrates that the pathogen burden would not have a large impact on the oxygen consumption in the conditions that we observed.

## 4 Discussion

Acceleration is widely applied as a metric of whole‐animal performance, which can be used to infer individual relationships between animals and their habitat (Brownscombe, Cooke, and Danylchuk 2017) as well as intra‐ and inter‐specific interactions (Barkley et al. 2020). As a highly dynamic variable in space and time, it was not surprising that all factors we expected to influence acceleration did indeed have clear effects. Acceleration progressively increased as days after release progressed. The analysis revealed clear activity relationships at the river level, likely related to the environmental contexts of the systems. For example, fixed effects for acceleration in Aurland were lower than for Vosso and Stjørdal; trout in Aurland were tagged in relatively slower flowing water and the river is colder than the others, which may have affected the overall activity of the fish as measured by the accelerometer (e.g., Dahlmo et al. 2023). Through all these significant relationships with acceleration, it was therefore notable that the relative infection burden was still a significant important factor explaining part of the variance in acceleration. Interestingly, activity metrics increased as fish were increasingly burdened by pathogens, suggesting a relationship between infection and behaviour. Higher levels of activity are known to yield faster metabolic rates, oxygen demand, and more rapid energy depletion (Gleiss, Wilson, and Shepard 2011; Halsey, Shepard, and Wilson 2011); for a fish like trout that is not feeding during the migration, this suggests a direct link between infection and several key fitness metrics such as energy conservation for spawning, overwintering survival, and lifespan if the impacts of pathogens persist.

Pathogenic bacteria, viruses and protists that infect fish can generate a systemic response that is captured by gene expression in gill tissue, as demonstrated here and validated in several previous studies (reviewed in Teffer and Miller 2019). Brown trout from Vosso, Stjørdal and Aurland were infected with several pathogens and the relative infection burden was used to capture total individual exposure to disease as per Bass et al. (2019). Measures of relative infection burden were similar to those for Atlantic salmon in Chapman et al. (2021). The operative pathogens in relative infection burden were typically *Ca*. Branchiomonas cysticola, *Ichthyobodo* spp. and *Flavobacterium psychrophilum* . Two of these, *Ca*. Branchiomonas cysticola and *F. psychrophilum* are commonly observed in salmonids in the Holarctic (e.g., Twardek et al. 2019; Chapman et al. 2021; Lennox et al. 2020) and may commonly be co‐infectious (Bass et al. 2017). *Ca*. Branchiomonas cysticola has recently been demonstrated to have a relationship with gill inflammation in saltwater (Wang et al. 2023); therefore, we considered copy numbers of this pathogen as a potential predictor of activity, however, there was no relationship and the model with relative infection burden was preferred as a composite analysis of infection on activity.

We do not know the exact mechanisms underlying the increased activity level of individuals with higher pathogen burden, but there are several examples of how pathogens can yield increased activity in their hosts. For example, Atkinson et al. (2018) showed that salmon lice infestation led to leaping behaviour of salmon, which clearly would increase the activity level of the fish. Although none of the pathogens are skin parasites, other behaviours to try to rid themselves of gill parasites/bacteria such as differential behavioural selection of freshwater or saline water are possible. Other mechanisms could be differential choices of thermal habitat (e.g., behavioural fever, Rakus, Ronsmans, and Vanderplasschen 2017), which could in theory lead to more activity due to suboptimal migration patterns. Pathogens may alternatively be associated with changes in equilibrium or stress that yields suboptimal behavioural patterns. The exact mechanisms are likely pathogen and disease specific, and most likely changes along the pathological progression of disease development. However, it is relevant to note that the changes in activity were not extreme.

While it appears counterintuitive that potentially compromised individuals would accelerate migration speeds, this relationship has been previously demonstrated in adult sockeye salmon returning to spawn in British Columbia, Canada. Migration speeds of sockeye salmon returning to spawn in the Fraser River were faster in both the marine and freshwater environments for fish that carried a ‘mortality related signature’, originally defined in Miller et al. (2011) and entered the river earlier than fish without this signature, but suffered elevated mortality after freshwater entry (Miller et al. 2011; Drenner et al. 2018). As semelparous species that die after spawning, it was hypothesised that enhanced stress and immune stimulation may have accelerated the drive of sockeye salmon to move towards spawning grounds before they died. A similar mechanism may be in place for iteroparous salmon and trout, where stress and disease may motivate fish to move more quickly to spawning grounds.

Although we only had three rivers and it was too few to identify consistent patterns at a landscape scale, it was interesting to note that trout from Vosso and Aurland had different infection burdens given their proximity and similar threats from open net‐pen fish farming in the fjords. A main difference among these three rivers is that Atlantic salmon is more abundant in Stjørdal than in Vosso and especially in Aurland, which may explain part of the variation in pathogen burden. This variation among rivers was particularly notable given that fish farming is not allowed in the Trondheimsfjord but is active in Sognefjorden (where Aurland is located), Osterfjorden and Sørfjorden (where Vosso is located), such that Aurland and Vosso fish are likely to contend with more pathogens emanating from the farms. Shea et al. (2020) did find elevated environmental DNA of salmonid pathogens around fish farms in western Canada, indicating that Vosso and Aurland would have been expected to be more exposed and burdened by pathogens than Stjørdal fish were. However, parts of Sognefjord and Sørfjorden are National Salmon Fjords and the areas affected by farming are quite distant from Aurland. Future intensification of fish farming in the north will propagate more pathogens in areas where fish may be less naturally adapted to coping with disease due to their evolutionary histories in colder areas where pathogens are not likely to replicate as readily (Dionne et al. 2007). However, our findings did not reflect increased pathogen burden on fish in areas with higher aquaculture intensity.

Our focus was on the marine life stage of adult sea‐run brown trout, which have already passed through key bottlenecks early in life that may greatly limit production. Juvenile life stages of brown trout and Atlantic salmon that migrate out of rivers into the fjord carry freshwater pathogens and encounter novel marine species that they must be able to overcome to reach the life stages at which we sampled. Sea lice is the most visible bottleneck for these young smolts and in western Norwegian fjords the lice is frequently reaching epidemic proportions during the smolt migration (Vollset et al. 2023). Some pathogens are common co‐infecting agents with sea lice, including *Paranucleospora theridion*, but this was surprisingly not common in our study despite Vosso and Aurland being close to fish farming and lice production risk areas. Other pathogens may be early‐life risk factors that should be studied in greater detail to understand performance of juvenile trout rather than returning adults. More comprehensive work on the impacts of pathogens on salmon smolt migrations has been conducted in the Pacific, including work indicating infection with the virus IHNV limits survival of migrating sockeye smolts (Jeffries et al. 2014). Notwithstanding, the concerns about pathogens emanating from fish farms in the migratory route of Atlantic salmon and sea‐run brown trout in the Atlantic suggest more molecular work should be conducted to contemplate the role of disease in early life stages of migrating salmon, when they are more likely to encounter disease agents for the first time. Future research should expand on our methodology to investigate performance impacts of pathogens on smolt behaviour and survival in both the freshwater and early marine migrations.

Despite identifying a relationship between the fish's infection burden and activity, the results should be viewed as a first indication of the role of pathogens on the marine migration of brown trout. The mensurative experimental design we used in aiming to investigate the role of disease in the ecology of wild fish are limited by several biases. Capturing wild fish necessarily limits the population to the catchable and available share within the stock, and we were therefore only able to sample and instrument fish that were vulnerable to our methods, which also differed slightly among locations. Pathogens can alter the performance and even cognition of animals such that they could be less prone to migration patterns that might normally coax them to swim into fyke or bag nets that were used in Vosso. Mixed gear sampling may be efficient to try to limit such biases but it is challenging to achieve a suitable sample of fish with all possible methods. We were only able to sample fish with sublethal infections at the time of capture, meaning that severely affected fish were likely left out due to survivor bias. The most heavily impacted fish from the stock are naturally absent, a bias that might make our results appear more favourable; however, it is not simple to overcome survivor bias in field studies. Experimental studies capturing and inoculating wild fish with pathogens could be sufficient to overcome some common biases, such as Wagner et al. (2005). Future investigators aiming to link wild fish health to performance by combining biopsies with tagging should aim to work with populations that have sufficient abundance to sample a relatively large share and should repeat sampling as much as possible across space and time.

## 5 Conclusions

The role of disease has long been of interest to ecology, but there has been relatively little research pairing tracking with infection and disease screening (Chretien et al. 2023), particularly with respect to performance ecology. Here, we found that acceleration‐derived activity of sea‐run brown trout was affected by the pathobiome after controlling for other factors we hypothesised would also affect activity such as days after tagging, time of day and river of origin. However, putting the changes in activity on a biological relevant scale by linking the acceleration data to oxygen consumption, it was not evident that the activity pattern had a large effect on oxygen consumption. Conservation of species like sea‐run brown trout that are affected by anthropogenic stressors like fish farming, which proliferates pathogens and results in frequent spillback, requires better knowledge of the infection rates of wild fish and the responses of these animals to such challenges. Our study demonstrates potential for the use of genomic tools in conservation for host and pathogen expression and we suggest that there will be increased use of these tools in coming years for supporting species conservation efforts.

## Author Contributions

**Robert J. Lennox:** conceptualization, investigation, funding acquisition, writing – original draft, methodology, validation, visualization, writing – review and editing, formal analysis. **Sindre H. Eldøy:** conceptualization, investigation, writing – review and editing, formal analysis. **Angela D. Schulze:** conceptualization, investigation, methodology, validation, writing – review and editing. **Kristina M. Miller:** conceptualization, investigation, writing – original draft, funding acquisition, writing – review and editing, visualization, methodology. **Trond Einar Isaksen:** conceptualization, investigation, writing – review and editing. **Jan G. Davidsen:** conceptualization, investigation, writing – review and editing, funding acquisition, writing – original draft, methodology. **Cecilie I. Nilsen:** conceptualization, investigation, writing – review and editing. **Lotte S. Dahlmo:** investigation, conceptualization, writing – review and editing. **Knut Wiik Vollset:** conceptualization, investigation, funding acquisition, writing – original draft, writing – review and editing, visualization, validation, methodology.

## Conflicts of Interest

The authors declare no conflicts of interest.

## Supporting information


**Data S1**.

**Data S2**.

## Data Availability

The data that support the findings of this study are available in the Supporting information of this article.

## References

[jfd14045-bib-0001] Akbarzadeh, A. , O. P. Günther , A. L. Houde , et al. 2018. “Developing Specific Molecular Biomarkers for Thermal Stress in Salmonids.” BMC Genomics 19: 1–28. 30326831 10.1186/s12864-018-5108-9 PMC6192343

[jfd14045-bib-0002] Akbarzadeh, A. , A. L. S. Houde , B. J. Sutherland , O. P. Günther , and K. M. Miller . 2020. “Identification of Hypoxia‐Specific Biomarkers in Salmonids Using RNA‐Sequencing and Validation Using High‐Throughput qPCR.” G3: Genes, Genomes, Genetics 10, no. 9: 3321–3336. 32694198 10.1534/g3.120.401487 PMC7466982

[jfd14045-bib-0041] Akbarzadeh, A. , D. T. Selbie , L. B. Pon , and K. M. Miller . 2021. “Endangered Cultus Lake Sockeye Salmon Exhibit Genomic Evidence of Hypoxic and Thermal Stresses While Rearing in Degrading Freshwater Lacustrine Critical Habitat.” Conservation Physiology 9, no. 1: coab089. 34858597 10.1093/conphys/coab089 PMC8633632

[jfd14045-bib-0003] Akbarzadeh, A. , T. Ming , A. D. Schulze , et al. 2024. “Developing Molecular Classifiers to Detect Environmental Stressors, Smolt Stages and Morbidity in Coho Salmon, *Oncorhynchus kisutch* .” Science of the Total Environment 951: 175626. 39168345 10.1016/j.scitotenv.2024.175626

[jfd14045-bib-0004] Atkinson, E. M. , A. W. Bateman , L. M. Dill , M. Krkošek , J. D. Reynolds , and S. C. Godwin . 2018. “Oust the Louse: Leaping Behaviour Removes Sea Lice From Wild Juvenile Sockeye Salmon *Oncorhynchus nerka* .” Journal of Fish Biology 93, no. 2: 263–271. 29956312 10.1111/jfb.13684

[jfd14045-bib-0005] Barkley, A. N. , F. Broell , H. Pettitt‐Wade , Y. Y. Watanabe , M. Marcoux , and N. E. Hussey . 2020. “A Framework to Estimate the Likelihood of Species Interactions and Behavioural Responses Using Animal‐Borne Acoustic Telemetry Transceivers and Accelerometers.” Journal of Animal Ecology 89, no. 1: 146–160. 31778207 10.1111/1365-2656.13156

[jfd14045-bib-0006] Bass, A. L. , S. G. Hinch , A. K. Teffer , D. A. Patterson , and K. M. Miller . 2017. “A Survey of Microparasites Present in Adult Migrating Chinook Salmon ( *Oncorhynchus tshawytscha* ) in South‐Western British Columbia Determined by High‐Throughput Quantitative Polymerase Chain Reaction.” Journal of Fish Diseases 40, no. 4: 453–477. 28188649 10.1111/jfd.12607

[jfd14045-bib-0007] Bass, A. L. , S. G. Hinch , A. K. Teffer , D. A. Patterson , and K. M. Miller . 2019. “Fisheries Capture and Infectious Agents Are Associated With Travel Rate and Survival of Chinook Salmon During Spawning Migration.” Fisheries Research 209: 156–166.

[jfd14045-bib-0008] Blem, C. R. 1980. “The Energetics of Migration.” In Animal Migration, Orientation, and Navigation, edited by S. A. Gauthreaux , 175–224. New York, NY: Academic Press.

[jfd14045-bib-0009] Bonneaud, C. , R. S. Wilson , and F. Seebacher . 2016. “Immune‐Challenged Fish Up‐Regulate Their Metabolic Scope to Support Locomotion.” PLoS One 11, no. 11: e0166028. 27851769 10.1371/journal.pone.0166028 PMC5113038

[jfd14045-bib-0010] Brownscombe, J. W. , S. J. Cooke , and A. J. Danylchuk . 2017. “Spatiotemporal Drivers of Energy Expenditure in a Coastal Marine Fish.” Oecologia 183: 689–699. 28093608 10.1007/s00442-016-3800-5

[jfd14045-bib-0011] Cáceres, M. D. , and P. Legendre . 2009. “Associations Between Species and Groups of Sites: Indices and Statistical Inference.” Ecology 90, no. 12: 3566–3574. 20120823 10.1890/08-1823.1

[jfd14045-bib-0012] Chapman, J. M. , L. A. Kelly , A. K. Teffer , K. M. Miller , and S. J. Cooke . 2021. “Disease Ecology of Wild Fish: Opportunities and Challenges for Linking Infection Metrics With Behaviour, Condition, and Survival.” Canadian Journal of Fisheries and Aquatic Sciences 78, no. 8: 995–1007.

[jfd14045-bib-0013] Chretien, E. , J. De Bonville , J. Guitard , et al. 2023. “Few Studies of Wild Animal Performance Account for Parasite Infections: A Systematic Review.” Journal of Animal Ecology 92, no. 4: 794–806. 36480357 10.1111/1365-2656.13864

[jfd14045-bib-0045] Dahlmo, L. S. , G. Velle , C. I. Nilsen , U. Pulg , R. J. Lennox , and K. W. Vollset . 2023. “Behaviour of Anadromous Brown Trout (*Salmo trutta*) in a Hydropower Regulated Freshwater System.” Movement Ecology 11, no. 1: 63. 37838718 10.1186/s40462-023-00429-7 PMC10576395

[jfd14045-bib-0040] Dhoubhadel, B. G. , M. Yasunami , L. M. Yoshida , et al. 2014. “A Novel High‐Throughput Method for Molecular Serotyping and Serotype‐Specific Quantification of *Streptococcus pneumoniae* Using a Nanofluidic Real‐Time PCR System.” Journal of Medical Microbiology 63, no. 4: 528–539. 24464695 10.1099/jmm.0.071464-0

[jfd14045-bib-0049] Dionne, M. , K. M. Miller , J. J. Dodson , F. Caron , and L. Bernatchez . 2007. “Clinal Variation in MHC Diversity With Temperature: Evidence for the Role of Host–Pathogen Interaction on Local Adaptation in Atlantic Salmon.” Evolution 61, no. 9: 2154–2164. 17767587 10.1111/j.1558-5646.2007.00178.x

[jfd14045-bib-0048] Drenner, S. M. , S. G. Hinch , N. B. Furey , et al. 2018. “Transcriptome Patterns and Blood Physiology Associated With Homing Success of Sockeye Salmon During Their Final Stage of Marine Migration.” Canadian Journal of Fisheries and Aquatic Sciences 75, no. 9: 1511–1524.

[jfd14045-bib-0014] Fry, F. E. J. 1947. “Effects of the Environment on Animal Activity.” Publications of the Ontario Fisheries Research Laboratory 68: 1–62.

[jfd14045-bib-0039] Gabrielsen, S. E. , R. J. Lennox , T. Wiers , and B. T. Barlaup . 2021. “Saltwater spawning Grounds of Sea‐Run Brown Trout (*Salmo trutta*) in Tidal Waters of a Major Norwegian River.” Environmental Biology of Fishes 104: 1207–1213.

[jfd14045-bib-0015] Gleiss, A. C. , R. P. Wilson , and E. L. Shepard . 2011. “Making Overall Dynamic Body Acceleration Work: On the Theory of Acceleration as a Proxy for Energy Expenditure.” Methods in Ecology and Evolution 2, no. 1: 23–33.

[jfd14045-bib-0016] Halsey, L. G. , E. L. Shepard , and R. P. Wilson . 2011. “Assessing the Development and Application of the Accelerometry Technique for Estimating Energy Expenditure.” Comparative Biochemistry and Physiology Part A: Molecular & Integrative Physiology 158, no. 3: 305–314. 10.1016/j.cbpa.2010.09.002 20837157

[jfd14045-bib-0017] Houde, A. L. S. , A. Akbarzadeh , O. P. Günther , et al. 2019. “Salmonid Gene Expression Biomarkers Indicative of Physiological Responses to Changes in Salinity and Temperature, but Not Dissolved Oxygen.” Journal of Experimental Biology 222, no. 13: jeb198036. 31209112 10.1242/jeb.198036 PMC6633282

[jfd14045-bib-0018] Houde, A. L. S. , O. P. Günther , J. Strohm , et al. 2019. “Discovery and Validation of Candidate Smoltification Gene Expression Biomarkers Across Multiple Species and Ecotypes of Pacific Salmonids.” Conservation Physiology 7, no. 1: coz051. 31620289 10.1093/conphys/coz051 PMC6788492

[jfd14045-bib-0019] Jeffries, K. M. , S. G. Hinch , M. K. Gale , et al. 2014. “Immune Response Genes and Pathogen Presence Predict Migration Survival in Wild Salmon Smolts.” Molecular Ecology 23, no. 23: 5803–5815. 25354752 10.1111/mec.12980

[jfd14045-bib-0020] Klaassen, M. 1996. “Metabolic Constraints on Long‐Distance Migration in Birds.” Journal of Experimental Biology 199, no. 1: 57–64. 9317335 10.1242/jeb.199.1.57

[jfd14045-bib-0036] Lennox, R. J. , J. M. Chapman , C. M. Souliere , et al. 2016. “Conservation Physiology of Animal Migration.” Conservation Physiology 4, no. 1: cov072. 27293751 10.1093/conphys/cov072 PMC4772791

[jfd14045-bib-0042] Lennox, R. J. , S. H. Eldøy , K. W. Vollset , et al. 2020. “How Pathogens Affect the Marine Habitat Use and Migration of Sea Trout (*Salmo trutta*) in Two Norwegian Fjord Systems.” Journal of Fish Diseases 43, no. 7: 729–746. 32364277 10.1111/jfd.13170

[jfd14045-bib-0021] Lennox, R. J. , S. H. Eldøy , L. S. Dahlmo , J. K. Matley , and K. W. Vollset . 2023. “Acoustic Accelerometer Transmitters and Their Growing Relevance to Aquatic Science.” Movement Ecology 11, no. 1: 45. 37501158 10.1186/s40462-023-00403-3 PMC10375738

[jfd14045-bib-0038] Lennox, R. J. , K. M. Miller , A. Madhun , et al. 2024. “The Pathobiome of *Salmo trutta* From the North Sea to the Barents Sea.” Journal of Biogeography.

[jfd14045-bib-0022] Livak, K. J. , and T. D. Schmittgen . 2001. “Analysis of Relative Gene Expression Data Using Real‐Time Quantitative PCR and the 2^−ΔΔCT^ Method.” Methods 25, no. 4: 402–408. 11846609 10.1006/meth.2001.1262

[jfd14045-bib-0023] Miller, K. M. , I. A. Gardner , R. Vanderstichel , et al. 2016. Report on the Performance Evaluation of the Fluidigm BioMark Platform for High‐Throughput Microbe Monitoring in Salmon, 1–293. Fisheries and Oceans Canada, Ottawa, Canada: Ecosystems and Oceans Science.

[jfd14045-bib-0047] Miller, K. M. , S. Li , K. H. Kaukinen , et al. 2011. “Genomic Signatures Predict Migration and Spawning Failure in Wild Canadian Salmon.” Science 331, no. 6014: 214–217. 21233388 10.1126/science.1196901

[jfd14045-bib-0024] Miller, K. M. , O. P. Günther , S. Li , K. H. Kaukinen , and T. J. Ming . 2017. “Molecular Indices of Viral Disease Development in Wild Migrating Salmon.” Conservation Physiology 5, no. 1: cox036. 28702195 10.1093/conphys/cox036 PMC5499884

[jfd14045-bib-0025] Mokhtar, D. M. , G. Zaccone , A. Alesci , M. Kuciel , M. T. Hussein , and R. K. Sayed . 2023. “Main Components of Fish Immunity: An Overview of the Fish Immune System.” Fishes 8, no. 2: 93.

[jfd14045-bib-0026] Oksanen, J. , G. Simpson , F. Blanchet , et al. 2022. “Vegan: Community Ecology Package. R Package Version 2.6‐4.” https://CRAN.R‐project.org/package=vegan.

[jfd14045-bib-0027] Palstra, A. P. , D. F. M. Heppener , V. J. T. Van Ginneken , C. Székely , and G. Van den Thillart . 2007. “Swimming Performance of Silver Eels Is Severely Impaired by the Swim‐Bladder Parasite Anguillicola Crassus.” Journal of Experimental Marine Biology and Ecology 352, no. 1: 244–256.

[jfd14045-bib-0043] Pedersen, E. J. , D. L. Miller , G. L. Simpson , and N. Ross . 2019. “Hierarchical Generalized Additive Models in Ecology: An Introduction With mgcv.” PeerJ 7: e6876. 31179172 10.7717/peerj.6876 PMC6542350

[jfd14045-bib-0028] R Core Team . 2024. R: A Language and Environment for Statistical Computing. Vienna, Austria: R Foundation for Statistical Computing. https://www.R‐project.org/.

[jfd14045-bib-0029] Rakus, K. , M. Ronsmans , and A. Vanderplasschen . 2017. “Behavioral Fever in Ectothermic Vertebrates.” Developmental & Comparative Immunology 66: 84–91. 27381718 10.1016/j.dci.2016.06.027

[jfd14045-bib-0030] Shea, D. , A. Bateman , S. Li , et al. 2020. “Environmental DNA From Multiple Pathogens Is Elevated Near Active Atlantic Salmon Farms.” Proceedings of the Royal Society B 287, no. 1937: 20202010. 33081614 10.1098/rspb.2020.2010 PMC7661312

[jfd14045-bib-0031] Teffer, A. K. , and K. M. Miller . 2019. “A Comparison of Nonlethal and Destructive Methods for Broad‐Based Infectious Agent Screening of Chinook Salmon Using High‐Throughput qPCR.” Journal of Aquatic Animal Health 31, no. 3: 274–289. 31343778 10.1002/aah.10079

[jfd14045-bib-0046] Twardek, W. M. , J. M. Chapman , K. M. Miller , et al. 2019. “Evidence of a Hydraulically Challenging Reach Serving as a Barrier for the Upstream Migration of Infection‐Burdened Adult Steelhead.” Conservation Physiology 7, no. 1: coz023. 31191906 10.1093/conphys/coz023 PMC6553125

[jfd14045-bib-0037] Vollset, K. W. , R. J. Lennox , J. G. Davidsen , et al. 2021. “Wild Salmonids are Running the Gauntlet of Pathogens and Climate as Fish Farms Expand Northwards.” ICES Journal of Marine Science 78, no. 1: 388–401.

[jfd14045-bib-0032] Vollset, K. W. , R. J. Lennox , H. Skoglund , et al. 2023. “Direct Evidence of Increased Natural Mortality of a Wild Fish Caused by Parasite Spillback From Domestic Conspecifics.” Proceedings of the Royal Society B 290, no. 1991: 20221752. 36695034 10.1098/rspb.2022.1752 PMC9880801

[jfd14045-bib-0033] Wagner, G. N. , S. G. Hinch , L. J. Kuchel , et al. 2005. “Metabolic Rates and Swimming Performance of Adult Fraser River Sockeye Salmon ( *Oncorhynchus nerka* ) After a Controlled Infection With Parvicapsula Minibicornis.” Canadian Journal of Fisheries and Aquatic Sciences 62, no. 9: 2124–2133.

[jfd14045-bib-0034] Wang, Y. , A. L. Bass , S. G. Hinch , et al. 2023. “Infectious Agents and Their Physiological Correlates in Early Marine Chinook Salmon ( *Oncorhynchus tshawytscha* ).” Conservation Physiology 11, no. 1: coad031. 37701371 10.1093/conphys/coad031 PMC10494280

[jfd14045-bib-0035] Wickham, H. 2016. ggplot2: Elegant Graphics for Data Analysis. New York: Springer‐Verlag.

[jfd14045-bib-0044] Wood, S. N. 2017. Generalized Additive Models: An Introduction With R. 2nd ed. New York, NY: Chapman and Hall/CRC. 10.1201/9781315370279.

